# An Epigenetic Regulator: Methyl-CpG-Binding Domain Protein 1 (MBD1)

**DOI:** 10.3390/ijms16035125

**Published:** 2015-03-05

**Authors:** Lu Li, Bi-Feng Chen, Wai-Yee Chan

**Affiliations:** 1 The Chinese University of Hong Kong—Chinese Academy of Sciences Guangzhou Institute of Biomedicine and Health Joint Laboratory on Stem Cell and Regenerative Medicine, School of Biomedical Sciences, Faculty of Medicine, The Chinese University of Hong Kong, Shatin, N.T., Hong Kong, China; E-Mail: s1155025779@cuhk.edu.hk; 2 The Chinese University of Hong Kong—Shandong University Joint Laboratory on Reproductive Genetics, School of Biomedical Sciences, Faculty of Medicine, The Chinese University of Hong Kong, Shatin, N.T., Hong Kong, China; E-Mail: cbifeng@whut.edu.cn; 3 Department of Biological Science and Biotechnology, School of Chemistry, Chemical Engineering and Life Sciences, Wuhan University of Technology, Wuhan 430070, Hubei, China

**Keywords:** methyl-CpG-binding domain protein 1, cancer, epigenetic regulation, transcriptional repression, nervous system

## Abstract

DNA methylation is an important form of epigenetic regulation in both normal development and cancer. Methyl-CpG-binding domain protein 1 (MBD1) is highly related to DNA methylation. Its MBD domain recognizes and binds to methylated CpGs. This binding allows it to trigger methylation of H3K9 and results in transcriptional repression. The CXXC3 domain of MBD1 makes it a unique member of the MBD family due to its affinity to unmethylated DNA. MBD1 acts as an epigenetic regulator via different mechanisms, such as the formation of the MCAF1/MBD1/SETDB1 complex or the MBD1-HDAC3 complex. As methylation status always changes along with carcinogenesis or neurogenesis, MBD1 with its interacting partners, including proteins and non-coding RNAs, participates in normal or pathological processes and functions in different regulatory systems. Because of the important role of MBD1 in epigenetic regulation, it is a good candidate as a therapeutic target for diseases.

## 1. Introduction

DNA methylation, a well-studied epigenetic modification, has been regarded as a key contributor to normal mammalian development and carcinogenesis [1,2]. A major approach to elucidate the mechanism of DNA methylation is to study proteins that directly interact with methylated DNA. Two families of protein, termed DNA methyltransferases (DNMTs) and methyl-CpG-binding domain (MBDs) proteins, have emerged as being associated with DNA methylation.

Three DNMTs have been shown to be involved in DNA methylation, namely DNMT1, DNMT3A and DNMT3B, respectively. The role of DNA methylation and DNMTs in mammalian development and carcinogenesis has been comprehensively studied and described [1,2,3,4,5,6]. Except for the donor of a methyl group to DNA, the modulator, which is able to bind methylated DNA and regulate the expression of gene, also has attracted much attention. In 1989, the first methyl binding protein (MBP), termed MeCP2, was discovered [7]. MeCP2 has a methyl-CpG-binding domain (MBD), which was the conserved domain of all MBD-containing proteins (MBD proteins) [8]. Currently, 11 MBD proteins are known, including STEDB1, STEDB2, BAZ2A, BAZ2B, MBD1, MBD2, MBD3, MBD4, MBD5, MBD6 and MeCP2 (NCBI Conserved Domain Database, CDD). CDD can be accessed at http://www.ncbi.nlm.nih.gov/Structure/cdd/cdd.shtml.

**Figure 1:**
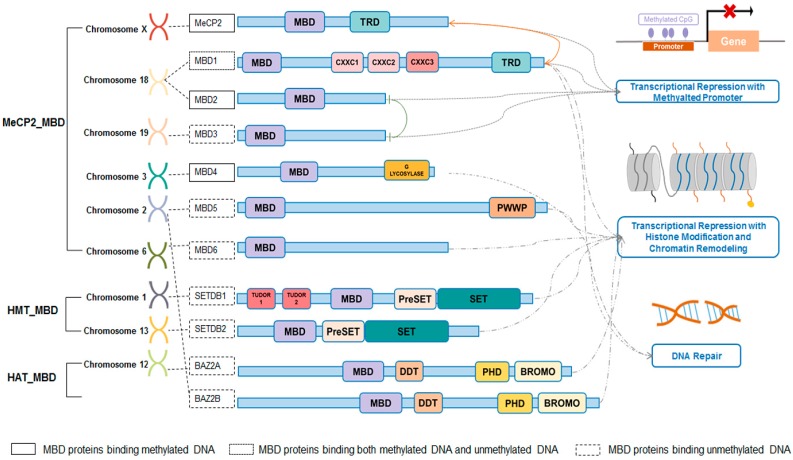
Locations, structures and functions of the methyl-CpG-binding domain MBD family.

Basically, the secondary structure of MBD is the same among all MBD proteins, but the sequence variations among them contribute to the differences in their properties and functions [9]. According to the sequence homology, the MBD superfamily can be divided into three groups, namely HMT_MBD, HAT_MBD and MeCP2_MBD, respectively. STEDB1, located in chromosome 1, and STEDB2, located in chromosome 13, belong to the histone methyltransferases (HMT_MBD) group, members of which share the MBD domain, PreSET domain and bifurcated SET domain [9,10,11]. With the property of protein lysine methyltransferase, both STEDB1 and STEDB2 are indicated to regulate transcriptional repression via the formation of heterochromatin [12,13,14]. The second group of the MBD superfamily is the histone acetyltransferases (HAT_MBD), including BAZ2A, located in chromosome 12, and BAZ2B, located in chromosome 2. They share the MBD domain, DDT domain, PHD-type zinc finger and the bromodomain. The bromodomain, which was the clue to identifying BAZ2A and BAZ2B, contributes to their participation in chromatin remodeling [9,10,11]. The remaining seven members, MeCP2 and MBD1-6 belong to the biggest group, the MeCP2_MBD group [9,10,11]. Members of the MeCP2_MBD group possess the “canonical” MBD domain, which contains an intron located at a conserved site [9,10,11,15]. The absence of “canonical” MBD disables HMT_MBD proteins and HAT_MBD proteins from binding to methylated oligonucleotides, generally distinguished by well-known MeCP2_MBD proteins, such as MBD2 [9,10,11]. Surprisingly, MBD3, MBD5 and MBD6, which own the “canonical” MBD, are not able to bind to methylated DNA, but colocalize with heterochromatin and contribute to transcriptional repression [11,16]. In many studies, MeCP2 and MBD1-4 were compared and contrasted, because of their overlapping functions in tumorigenesis and development [17]. Generally, MeCP2, MBD2 and MBD3 are involved in gene silencing associated with methylation of the promoter; MBD1 primarily modifies histone and contributes to the formation of heterochromatin, and MBD4 takes part in DNA repair [8]. However, sometimes, they form complexes and function in other biological processes. More details about the relationship and functions of MeCP2_MBD proteins will be discussed in the “Specificity and Redundancy of MeCP2_MBD Proteins” section. The locations and structures of MBD proteins and the epigenetic regulations that they are involved in are shown in Figure 1 [8,18].

In the MBD family, MBD1 stands out because of its unique structure and specific function in gene regulation, although it has not been studied very well compared to MBD2 and MeCP2 so far. Besides regulating epigenetic change via DNA methylation, MBD1 offers another way of epigenetic regulation by modulating the H3K9me3 histone mark. MBD1 has three specific CXXC domains distinct from other MBD proteins. The first two CXXC domains (CXXC1 and CXXC2) allow MBD1 to bind to methylated DNA, but the presence of the third CXXC domain (CXXC3) enables MBD1 to occupy unmethylated DNA [19]. The dual-affinity to both methylated DNA and unmethylated DNA enables MBD1 to act in different epigenetic regulations, such as methylation of the gene promoter and modulation of histone. Furthermore, recent studies showed important roles of MBD1 in cancer and neuron development [20,21,22,23,24,25,26,27,28,29,30,31,32,33,34,35,36]. These features make MBD1 a novel target for studying different epigenetic regulations performed by a single protein, for understanding the mechanism of how to associate DNA methylation with histone modification and potentially offer a new therapeutic target for cancer and neuron diseases.

## 2. Methyl-CpG-Binding Domain Protein1 (MBD1) Protein

**Figure 2:**
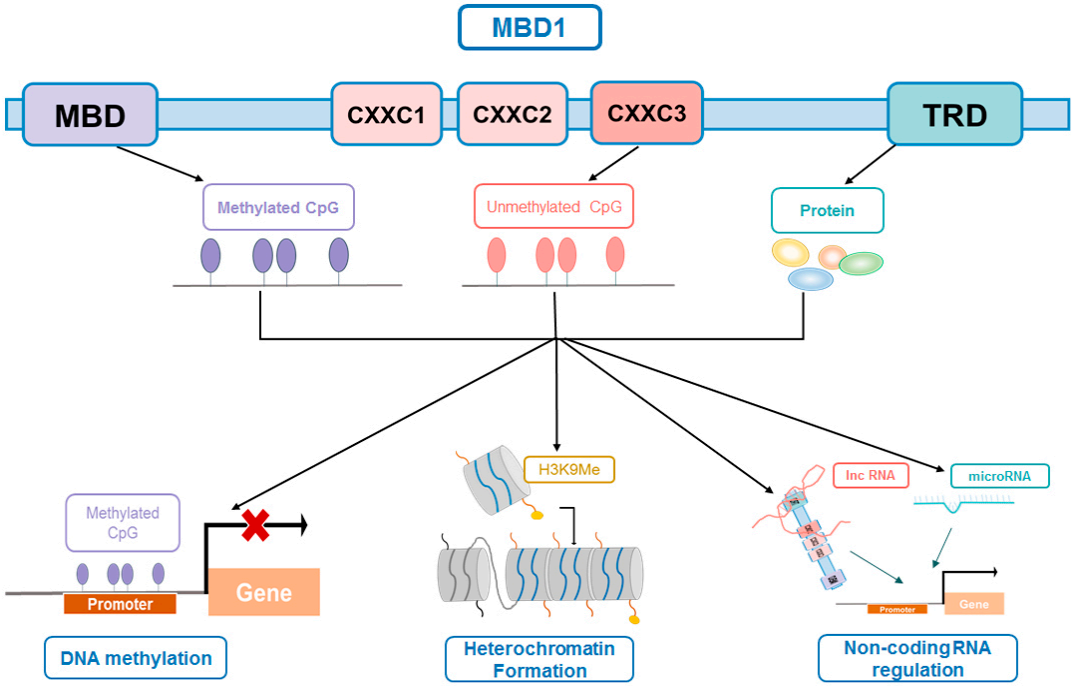
Structure of methyl-CpG-binding domain protein1 (MBD1) and the basic mechanism of MBD1 epigenetic regulation.

As the largest member of the MBD family, MBD1 has a unique and complex structure distinct from other MBD members. Taking human MBD1 protein as an example (Figure 2), except for the conserved MBD domain at its *N*-terminus, it also has a transcriptional repression domain (TRD) at its *C*-terminus [37]. Both domains are related to the interaction between MBD1 and other proteins; however, the MBD domain guides the binding of MBD1 to methylated DNA, and the TRD domain regulates transcriptional repression. As the common domain of the entire MBD family, the MBD domain of MBD1 executes the basic function of recognizing the methyl group at the methylated DNA site through a hydrophobic patch, which consists of five highly-conserved MBD amino acid residues [38]. This structure facilitates MBD domain access to the methyl group located in the major groove of nucleosomal DNA, but avoids the interference of the core histone protein located in the minor groove or inward-facing DNA backbone [38,39]. DNA binding forces MBD to form α helix and β sheet to embed into the major groove of DNA. These two layers with the rearranged loops comprise the DNA interface.

Because of alternative splicing events, MBD1 also has two or three CXXC-type zinc finger domains; the number of CXXC motifs varies among MBD1 isoforms and depends on whether MBD1 binds to unmethylated DNA [19]. Such variation has been found both in human and mouse cells [19,40]. The MBD1 isoforms containing the first two CXXC motifs can only bind to methylated DNA; however, the third CXXC motif, termed CXXC3, enables MBD1 binding to DNA irrespective of its methylation status [41]. In brief, all three CXXC motifs share high similarity, but the first two CXXC motifs are different from CXXC3 in key amino acids located in the positively-charged DNA-binding loop, which disables them from binding DNA [19]. MBD1 isoforms that carry CXXC3 can repress transcription from both methylated and unmethylated promoters, but the MBD1 isoforms without CXXC3 can only silence the transcription of highly methylated genes [42]. Briefly, when the genome is drastically unmethylated, like in preimplantation mouse embryos, CXXC3 facilitates the binding of MBD1 to target genes [43,44]. A more recent study in mouse embryonic stem cells (ESC) showed that CXXC3 only binds to unmethylated DNA when the MBD domain is completely deleted. Even mutation in the MBD domain or deletion of the methylation of DNA cannot induce recruitment of MBD1 to unmethylated DNA, which is in contrast to a previous observation in fibroblast, indicating that MBD1 acts differently in different cell types [19,45].

Although the CXXC3 motif allows MBD1 binding to unmethylated DNAs and silences their transcription, the MBD domain is reported as the predominant and critical domain in MBD1 in most cases, considering that the genome is generally methylated [46]. In Dnmt1, Dnmt3a and Dnmt3b triple-knockout embryonic stem (ES) cells, the methylation of DNA is totally lost, and MBD1 cannot localize to CpG islands that are normally methylated in wild-type ES cells [45]. This result indicated that methylation of DNA is the prerequisite for localization of MBD1 to targeting of the CpG island. The MBD domain of MBD1 preferentially binds to TCMGCA and TGCMGCA, but the CXXC3 motif does not have such affinity to any specific sequence, except for a single CpG when it binds to unmethylated DNA. Interestingly, the high affinity between the MBD domain and TCMGCA/TGCMGCA only exists in the binding of MBD1 to methylated DNA, but not between MeCP2 and methylated DNA, indicating a unique DNA reading behavior of the MBD domain of the MBD1 protein. In the transient reporter assay using fusion protein, the MBD domain, but not CXXC3, was shown to be the essential domain inducing stable binding of MBD1 to its target genes. Moreover, during differentiation from ES cells to neural progenitor cells, the increase of recruitment of MBD1 and the growing level of DNA methylation are consistent [45]. In sum, the CXXC motifs distinguish MBD1 from other MBD proteins, but the MBD domain endows MBD1 the substantial characteristic of methyl-DNA binding and functioning as a transcriptional repressor. In addition, the CXXC3 domain both supports unmethylated DNA binding and behaves as a cooperator that stabilizes the binding between MBD1 and methylated DNA.

## 3. MBD1 and Epigenetic Regulation in Cancer

### 3.1. DNA Methylation, Heterochromatin Formation and Transcriptional Repression

DNA methylation and heterochromatin condensation are known to induce transcriptional repression. Numerous studies indicated the importance of aberrant DNA methylation and heterochromatin formation in cancer [47,48]. The tumorigenesis may be caused by MBD1 recruiting transcriptional repressor proteins and triggering the silencing of tumor suppressor genes. MBD1, which specifically binds to methylated DNA, can induce histone methylation at lysine residue 9 of histone H3 (H3K9me) and, therefore, builds a linkage between two epigenetic modifications. For instance, it recruits histone methyltransferase SETDB1 through its TRD domain, together with MBD1-containing chromatin associated factor 1 (MCAF1), to form the MCAF1/MBD1/SETDB1 complex [49,50,51]. During DNA replication, this complex aids in forming H3K9me with the help of heterochromatin protein 1 (HP1) and promotes the formation of condensed heterochromatin [49,50,51]. Participation in heterochromatin formation indicates that MBD1 is a histone modulator and a chromatin assembler. A recent study suggested that this complex is also critical for the maintenance of X chromosome inactivation in female mammalian cells [52]. Covalent attachment of small ubiquitin-related modifiers (SUMOs) to MBD1, termed SUMOylation of MBD1, can be regulated by protein inhibitors of activated STAT1 (PIAS1) and PIAS3 E3 SUMO-ligase [53]. Uchimura *et al.* [54] reported that SUMOs promoted the formation of heterochromatin by facilitating the recruitment of SETDB1 to MBD1 through MCAF1. However, Lyst *et al.* [53] claimed that the SUMOylation of MBD1 might destabilize the interaction between MBD1 and SETDB1. These two cases suggested that the effect of the SUMOylated MBD1 system might depend on cell lines and culture conditions. However, the importance of SUMOylated MBD1 and SETDB1 has been confirmed in transcriptional repression [53,54].

Except for the MCAF1/MBD1/SETDB1 complex, MBD1 is also involved in other mechanism of regulating heterochromatin formation. It can bind to polycomb group (PcG) proteins via the CXXC domains to silence the *HOXA* gene [55]. In HeLa cells, MBD1 and PcG were found in the same heterochromatin foci. Further study showed that they share a partially redundant function in heterochromatin formation and transcriptional silencing [55]. Moreover, MBD1 can be recruited to its target promoter by PML-RARα through an HDAC3-mediated mechanism in acute promyelocytic leukemia [56]. The MBD1-HDAC3 complex contributed well to the condensation of chromatin and the maintenance of transcriptional repression [56]. MBD1 together with other repressor proteins, such as 3-methyl purine DNA glycosylase (MPG), was shown to bind to the methylated gene promoters to form a tight repressor complex [57]. Moreover, the MBD1-MPG complex was indicated to repair DNA damage [57]. Interestingly, during heterochromatin formation, the *C*-terminus of MBD1 (MBD1-c) containing the TRD domain binds to different protein components in different manners [51]. MCAF1 and MPG can bind to both the *N*-terminus and *C*-terminus of MBD1-c, but HDAC3 has higher affinity to the *C*-terminus of MBD1-c. By combination of hydrophobic and charge-charge interactions, MBD1 distinguishes its different components and is capable of regulating a complex network in transactional repression [51]. The epigenetic regulation potentially related to MBD1 and cancer is shown in Table 1.

**Table 1:** Regulatory mechanisms and cancer types related to MBD1.

Epigenetic Regulation Type	Component	Target Genes	Cancer Types
Histone methylation (heterochromatin formation)	MCAF1/SETDB1 PML-RARα/HDAC3 MPG	-	Acute promyelocytic leukemia
Histone methylation (heterochromatin formation)	PcG	*HOXA*	-
Histone methylation (heterochromatin formation) lnc RNA	*H19* lnc RNA	*Igf2*, *Slc38a4*, *Dcn*, *Dlk1*, *Peg1*	-
SUMOylation of MBD1	PIAS1/PIAS3 E3 SUMO-ligase	-	-
DNA methylation	-	*CDH1*, *RASSF1A*, *TIMP3*, *P14ARF*, *Rb*	Pancreatic cancer
DNA methylation	Twist/SIRT1	*CDH1*	Pancreatic cancer
Loss of heterozygosity	-	-	Prostate cancer
DNA mutation	-	-	Colon cancer/lung cancer
DNA methylation	PIAS1	IFN-γ, pSTAT1	Colon cancer
Polymorphism	-	-	Lung cancer

### 3.2. Tumorigenesis

The hypermethylation of promoter of the tumor suppressor gene is a well-characterized event in tumorigenesis [58]. Since MBD1 is a transcriptional regulator that binds methylated CpG islands of tumor suppressor genes and represses their transcription, it was strongly suggested to promote tumorigenesis. Indeed, in pancreatic cancer (PC), knocking down MBD1 using small interfering RNA (siRNA) would dramatically inhibit cell growth and invasion, induce apoptosis, as well as increase the sensitivity of cells to radiation and cisplatin [20,21,22]. More detailed studies revealed that inhibition of MBD1 upregulated the expression of several tumor suppressor genes related to DNA methylation, including CDH1, RASSF1A, TIMP3, P14ARF and Rb [23]. In addition, the function of MBD1 in mediating chemo-radio-resistance is attributed to its regulation of MDC1 (mediator of DNA damage checkpoint protein 1) and NBS1 in the presence of DNA damage repair [22]. More importantly, MBD1 was associated with Twist and NAD-dependent deacetylase sirtuin-1 (SIRT1) [20]. They form the Twist-MBD1-SIRT1 complex on the CDH1 promoter, which resulted in reducing E-cadherin transcription activity and increasing cell EMT ability. This transcriptional repression provided the molecular mechanism underlying MBD1-promoted PC invasion and metastasis. On the other hand, higher MBD1 expression is correlated with lymph node metastasis and poor survival in PC patients [20]. Taken together, MBD1 may serve as a potential therapeutic target for PC. Interestingly, the delivery of RNA interference of MBD1 into pancreatic cancer cells *in vitro* by poly(d,l-lactic-*co*-glycolic acid) (PLGA)-poloxamer nanoparticles had been tested and showed a good therapeutic effect [21].

The situation was not quite the same in prostate cancer. Depletion of MBD1 had no effect on the proliferation and apoptosis of prostate cancer cells. mRNA expression profiling showed that the expression of the vast majority of genes in both control and MBD1-deletion prostate cancer cells remained unchanged [22]. Surprisingly, deletion of MBD1 enhanced the invasion and migration of the prostate cell line. Although previous studies pointed to an oncogenic role of MBD1 in cancer, the evidence was that MBD1 was located on chromosome 18q21, a region of frequent loss of heterozygosity in several cancers, suggested that MBD1 might represent a candidate tumor suppressor gene [25]. Mutation analyses revealed that a number of mutations occurred in the MBD1 gene in colon and lung cancer cell lines, indicating a suppressor role of MBD1 in human tumorigenesis [26]. A systematic study of colon carcinoma indicated that MBD1 and PIAS1 were located at the methylated promoter of the IRF8 gene and inhibited the activation of IFN-γ, resulting in the repression of pSTAT1 [59]. On the other hand, association studies on polymorphisms of the MBD1 gene with human cancers revealed that MBD1 SNPs were not significantly associated with breast cancer, while two polymorphisms (rs125555 and rs140689) of MBD1 were significantly correlated with lung cancer risk, which supported the evidence that MBD1 contributes to the risk of cancer [27,28]. Taken together, these studies suggest that MBD1 may play dual roles in human tumorigenesis, via interaction with different proteins. The mechanism of epigenetic regulation related to MBD1 offers much information about cancer diagnosis and drug development. However, further elucidation of the expression of MBD1 in different cancer types or different stages is required. Its critical role in epigenetic regulation in cancer suggests it to be a good target for cancer treatment.

## 4. MBD1 and Epigenetic Regulation in the Nervous System

Although MBD1 is expressed ubiquitously in various tissues, its high expression level in neural stem cells (NSCs) and neurons makes it an important protein in the nervous system [29]. Another piece of evidence of its critical role in the nervous system is that, in spite of the fact that MBD1[−/−] mice had no obvious developmental defects and lived healthily, they had trouble in learning behavior, with decreased neurogenesis in the hippocampus [29]. The link between MBD1 and autistic symptoms in both mouse and human emphatically highlights its function in the brain [30,31]. Studies in the MBD1 double-knockout (−/−)mice showed that a serotonin receptor, Htr2c, was regulated by MBD1 [30]. Since the serotonin system was reported to relate to autism, the regulation of MBD1 on Htr2c is likely to contribute to autism [32].

Epigenetic regulation, including DNA methylation, histone methylation and non-coding RNAs, are highly correlated and indicated to be involved in developmental processes [33,60]. Many researchers focus on studying the correlation between methylation and non-coding RNAs in development processes, such as neuron development. Zhao’s group [29,30] studied the role of MBD1 in NSC differentiation and proliferation and found that its loss led to NSC differentiation deficiency. Both DNA methylation and miRNAs are involved in MBD1-related NSC differentiation and proliferation. In 2008, they reported that NSCs derived from MBD1−/− mice were hypomethylated in the promoter of basic fibroblast growth factor 2 (Fgf-2), which could promote NSC proliferation and maintenance of undifferentiated NSCs [33]. Moreover, inhibition of MBD1 in adult NSCs resulted in decreased neuronal differentiation [33]. MBD1 binds to the promoter of Fgf-2, leading to hypermethylation of the Fgf-2 promoter and repressing the expression of Fgf-2 in NSCs. This resulted in normal neuronal differentiation [33]. In 2010 and 2013, respectively, the same group reported a novel mechanism involving MBD1 and miRNA in nervous system development. MicroRNA (miRNA) is a kind of small, non-coding RNA comprised of ~22 nucleotides. Generally, it post-transcriptionally regulates the expression of target genes via perfectly or imperfectly binding to the 3' untranslated region of the target mRNA [61]. Zhao’s group [34] claimed that several miRNAs, especially miRNA-184 and miRNA-195, were directly inhibited by MBD1 in the balance of NSC proliferation and differentiation. A high expression level of miR-184 increased NSC proliferation, and inhibition of miR-184 could rescue the symptom of MBD1 deficiency, including the lack of NSC differentiation [34]. Numblike, the downstream regulator of miR-184, was indicated to be critical for brain development and involved in the MBD1/miR-184 regulating system. No downstream regulator of miR-195 in the MBD1 regulating system has been reported. However, MBD1 and miR-195 form a regulatory loop in NSC differentiation and proliferation [35]. Except for the repression of MBD1 on miR-195, MBD1 itself is also regulated negatively by miR-195 [35]. Further, *H19*, a long non-coding RNA (lnc RNA), can also form a complex with MBD1, which will interact with histone lysine methyltransferases, SETDB1 and SUV39H1, brings transcriptionally repressive marker, H3K9me3, to the differentially methylated region (DMR) of imprinted genes and, consequently, inhibits the expression of these genes. The *H19*-MBD1 complex was reported to repress the expression of five genes (*Igf2*, *Slc38a4*, *Dcn*, *Dlk1* and *Peg1*) in an imprinted gene network (IGN), which was important for embryonic development [36]. This regulatory system perfectly and completely involves all three epigenetic regulations and highlights the central role of MBD1 in a precisely complex network, indicating MBD1 as a critical connector and regulator in the epigenetic regulation system. The epigenetic regulations potentially related to MBD1 and diseases of the nervous system are shown in Table 2.

**Table 2:** Regulatory mechanisms and diseases of the nervous system related to MBD1.

Epigenetic Regulation Type	Component	Target Genes	Disease Types
DNA methylation	-	*Htr2c*	Autism disease
DNA methylation	-	*Fgf-2*	Neurogenesis
miRNA	miR-184	*Numblike*	Neurogenesis
miRNA	miR-195	*miR-195/MBD1*	Neurogenesis

## 5. Specificity and Redundancy of MeCP2_MBD Proteins

As the initial members of the MBD family, MBD1-4 and MeCP2 were always studied as a cluster without the other members. A recent study that tried to elucidate the relationship between MeCP2_MBD proteins and DNA methylation indicated that MBD1, MBD2, MBD4 and MeCP2, but not MBD3, shared the same binding methylated site in the promoter of genes, like *Peg10* and *Xist*, in mouse ES cells [45]. In human cancer, different MeCP2_MBD proteins can also bind the methylated promoter of some genes. For example, MBD2 and MBD4 can bind the methylated promoter of P16^INK4a^, while MeCP2, MBD1 and MBD2 can bind to that of CDH1 [62]. Other studies also reported similar behavior of binding to methylated DNA shared by MeCP2_MBD proteins, a tendency to bind DNA with s higher methylation density [45]. Different from DNMT-deficient mice dying at an early development stage, knocking out MBD1, MBD2 and MeCP2 proteins respectively in mice was not lethal. These three animal models showed much milder, but distinctive symptoms, suggesting a potential redundancy among these MeCP2_MBD proteins [29,30,63,64,65]. In many cases, although they have the “canonical” MBD domain, neither their expression nor their behavior is identical. MeCP2 and MBD1-4 proteins are expressed in all murine somatic tissues, but with different expression levels [15]. For example, the expression of MBD1 and MBD3 is much higher than MBD2 and MBD4 in brain. MBD4 expresses at a relatively lower level compared to MBD1-3 in all somatic tissues. Their expression in ESC is also varied. Only MBD3 expresses highly in ESC, while MBD2 and MBD4 are expressed extremely lowly, and MeCP2 and MBD1 are totally absent [15]. Given the indispensable nature of methylation in ESC, the loss of MeCP2_MBD proteins in ESC is not surprising [15,66]. Because of two alterations presented in the MBD domain, MBD3 does not bind to methylated DNA, and its expression being unaffected by the methylation status in ESC is reasonable [67]. Except for the variation of the expression level in different somatic tissues or ESC, MeCP_MBD proteins also differ in affinity toward methylated DNA. Among MeCP2 and MBD1-6, MBD2 has the highest affinity toward methylated DNA and the widest binding profile; MBD1, MBD4 and MeCP2 are lower in affinity toward methylated DNA, while MBD3, MBD5 and MBD6 do not bind at all [68]. A possible explanation of this variation in methylated DNA affinity is the different requirement for the base composition near methyl-CpG. For example, MeCP2 primarily binds to DNA containing enriched A/T bases flanking methyl-CpG; MBD1 has a preference toward TCMGCA/TGCMGCA, but MBD2 has no requirement for binding sequences [45,68]. In the case of MBD4, the presence of the TGD domain results in its preference for the TG:meCG mismatch and enable MBD4 to repair this mismatch by glycosylation [69]. It can be assumed that the specific domains other than the MBD domain increase the specificity of the MeCP_MBD proteins.

In most of the cases, each of the MeCP2_MBD proteins has numerous specific targets and is associated with different cancer types [62]. For instance, in a study of the differential expression of MeCP2_MBD proteins among 10 cancer cell lines, MBD1 showed the highest expression level in three colon cancer cell lines, MBD2 was expressed the highest in the Raji cell line (leukemia) and the MDA-MBD-231 cell line (breast cancer), in which MeCP2 was drastically reduced [62]. However, subsequent studies showed that the expression level of MeCP2_MBD proteins was not related to their preferential use of the promoter in different cancer types. Instead, the interaction between the transcriptional repression complexes that they were involved in and the specific cell-type nuclear factors potentially contributed to their selection of the binding promoters [62]. Another reasonable explanation of their specificity in targeting genes is their requirements for binding sequences. Because of the sequence variations among promoters, different MeCP2_MBD proteins will selectively bind their specific targets. Thus, the differential expression of the targeted genes in different types of cancer partially decides their cancer correlation. Combining these facts, it is clear that the MeCP2_MBD proteins have partially overlapping functions, but still retain their own properties in cancer [46].

## 6. Therapeutic Application

It is known that MBD1 can bind to aberrant methylated promoters and dysregulate gene expression. As MBD1 has a distinct binding profile in specific cancer types, it has been used to identify novel methylated genes that are the potential targets for cancer therapy [67]. The predominant domain of MBD1 that interacts with miRNAs can be used to find more MBD1-regulated miRNAs and downstream proteins. Because the MBD1-miRNA regulatory system has been indicated to be important for neural stem cell fate determination, studying this system can extend the knowledge of normal brain development and allow the search for specific miRNAs as therapeutic candidates for brain diseases, such as autism.

The abnormal methylated status in the genome is the hallmark of cancer. So far, therapy aiming at aberrant methylation of cancer uses 5-aza-(2-deoxy)cytidine to inhibit the functions of DNA methyltransferase and to erase the abnormal methylation of DNA. The combination of 5-aza and HDAC inhibitors was applied in the treatment of hematological malignancies [8,67]. However, the concern about using 5-aza is its nonspecific demethylating effect, which may potentially activate methylated “oncogenes” by demethylating promoters and result in chromatin instability by inducing global hypomethylation. Instead, reagents targeting MBD proteins, such as MBD1, are better candidates for cancer therapy. As discussed before, MBD1 recognizes methylated DNA and induces chromatin remodeling, indicating that it regulates transcription by decoding methylated DNA rather than inducing DNA methylation. In addition, MBD protein is known to regulate specific genes and to be involved in different cancer types, whereas the cancer types that MBD1 is associated with and the underlying mechanisms remain to be clarified. Combining its functions and distinct binding profile in cancers, the reagents, such as hairpin RNA, that relieve methylated DNA from the binding of MBD1 may be sufficient to break the linkage between DNA methylation and transcriptional repression without a change in the methylation status or chromatin re-organization. In other words, if the information offered by methylated DNA cannot be recognized, the aberrant DNA status may be meaningless. Moreover, considering the critical function of the TRD domain in tumorigenesis, a potential therapy for cancer is to investigate the usefulness of the inhibitor targeting the TRD domain specifically.

## 7. Conclusions and Prospects

In summary, the MBD1 protein predominantly acts as a transcriptional repressor by recruiting various proteins or non-coding RNAs to maintain the methylation of the transcriptionally-repressive marker, H3K9me3. In the past few years, its dual role in cancer and regulatory function in neural development has been confirmed. These findings light the way toward investigating its involvement in epigenetic regulation. With more binding partners of MBD1 discovered in the future, it will significantly expand our knowledge about the mechanisms of gene regulation.

The nonlethal effect of knocking out MBD1, MBD2 and MeCP2 individually in mice suggests their compensatory role in development [29,30,63,64,65]. Sharing the binding position of methylated DNA may partially explain the compensating effects. However, how they compensate for each other is unclear. Conditionally knocking out each member of the MBD family at a specific developmental stage may decipher the compensating mechanism. Specific to MBD1, further study about its functions and biological significance in development is necessary. The structure of the TRD domain and the interaction between the TRD domain and transcriptional suppressor proteins are not well studied. Because the regulatory loop including MBD1 and miRNAs has been found, further study about which domain of MBD1 participates in the regulation will enhance our understanding of this regulatory system. As discussed before, except in the neuronal system, MBD1 seems to be indispensable in normal development. This is contrary to the important role of Dnmts and SETDB1 in development [8]. Based on the specific effect on the neural system, it is possible that MBD1 systematically regulates lineage specification, rather than supporting normal development. An experiment of knocking out MBD1 at the developmental stage related to certain lineage specification may verify this hypothesis. On the other hand, it is known that MBD1 primarily acts as a transcriptional suppressor. How it becomes an integral part in different complexes that regulate different pathways is under investigation. For instance, the mechanism of how the complexes, such as MCAF1/SETDB1/MBD1 and MBD1/PML-RARα/HDAC3, function in the replication of chromatin remains unexplored [55,70]. Additionally, understanding the mechanism of how the chromatin remodeling complexes containing MBD1 are epigenetically regulated, such as the SUMOylation of the MCAF1/SETDB1/MBD1 complex, will be a promising way to study how the linkage between epigenetic regulation and the organization of the chromatin structure is constructed. The CXXC3 domain of MBD1 contributes to its affinity to unmethylated DNA. Given that global hypomethylation is a characteristic of cancer, but being studied less than methylation of the promoter, studying the regulation system of MBD1 and hypomethylated DNA will be important in unveiling a novel regulatory mechanism in carcinogenesis.
